# Supplementary material for: Integrating genomic structural equation modeling and experimental validation to unravel the genetic basis of male genital lichen sclerosus
Source: Front Immunol. 2026 Jul 16;17:1857945. doi: 10.3389/fimmu.2026.1857945 (PMC13435250; doi:10.3389/fimmu.2026.1857945)
Supplement: Supplementary file 6 [file SupplementaryFile5.zip › result/fuma_PSYCH_factor_sCCA2_focus.chr6.chr6.31571218.32682664.pop1.pdf]

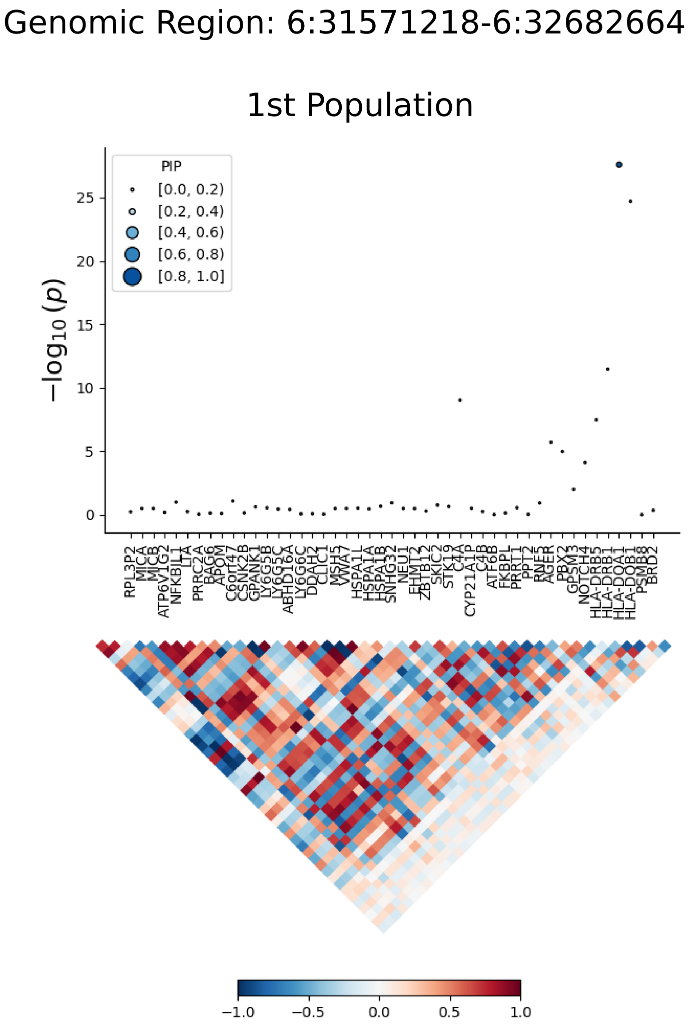
Genomic Region: 6:31571218-6:32682664
1st Population
PIP
[0.0, 0.2)
[0.2, 0.4)
[0.4, 0.6)
[0.6, 0.8)
[0.8, 1.0]
$-\log_{10}(p)$
0
5
10
15
20
25
RPL3P2
MICA
MICB
ATP6V1G2
NFKBIL1
LTA
PRRC2A
BAG6
APOM
C6orf47
CSNK2B
GPANK1
LY6G5B
LY6G5C
ABHD16A
LY6G6C
DDAH2
CLIC1
MSH5
VWA7
HSPA1L
HSPA1A
HSPA1B
SNHG32
NEU1
EHMT2
ZBTB12
SKIC2
STK19
C4A
CYP21A1P
C4B
ATF6B
FKBPL
PRRT1
PPT2
RNF5
AGER
PBX2
GPSM3
NOTCH4
HLA-DRB5
HLA-DRB1
HLA-DQA1
HLA-DQB1
PSMB8
BRD2
−1.0
−0.5
0.0
0.5
1.0
